# Fruit ripening in *Lycium barbarum* and *Lycium ruthenicum* is associated with distinct gene expression patterns

**DOI:** 10.1002/2211-5463.12910

**Published:** 2020-06-29

**Authors:** Jianhua Zhao, Haoxia Li, Yue Yin, Wei An, Xiaoya Qin, Yajun Wang, Yunfang Fan, Yanlong Li, Youlong Cao

**Affiliations:** ^1^ Wolfberry Engineering Research Institute Ningxia Academy of Agriculture and Forestry Sciences/National Wolfberry Engineering Research Center Yinchuan China; ^2^ Desertification Control Research Institute Ningxia Academy of Agriculture and Forestry Sciences Yinchuan China

**Keywords:** arogenate dehydratases, BAM1, GATA22, HAT5, PED1, SCL32

## Abstract

Goji berries have been used as food and medicine for millennia. Due to their high morphological similarity, fruits of two distinct species belonging to the family Solanaceae, *Lycium barbarum* (LB) and *Lycium chinense* (Chinese boxthorn), are usually marketed together as goji berries, but nearly 90% of all commercially available goji berries belong to the former species. A third closely related species, a wild perennial thorny shrub native to north‐western China, *Lycium ruthenicum* (LR; known as Russian box thorn, and its fruit as black wolfberry), has become a popular choice for combating soil desertification and for alleviating soil salinity/alkalinity due to its high resistance to the harsh environment of saline deserts. Despite the phylogenetic closeness of LB and LR, their fruits are very different. To identify the genes involved in these distinct phenotypes, here we studied expression patterns of 22 transcriptional regulators that may be crucial drivers of these differences during five developmental stages. *BAM1* may contribute to higher sugar content in LB. High expression of *BFRUCT* in ripe LR is likely to be an evolutionary adaptation to fruit ripening in an arid environment. Two arogenate dehydratase paralogues*, CHS* and *LDOX*, are probably crucial elements of the mechanism by which LR accumulates much higher levels of anthocyanin. *DXS2* (carotenoid accumulation in LB) and *CCD4* (carotenoid degradation in ripe LR fruit) may be crucial drivers behind the much higher content of carotenoids in LB. *EIL3* and *ERF5* are two transcription factors that may contribute to the higher abiotic stress resilience of LR. *GATA22*‐like appears to have more important roles in growth than ripening in LB fruit and vice versa in LR. *HAT5*‐like exhibited opposite temporal patterns in two fruits: high in the 1st stage in LB and high in the 5th stage in LR. *PED1* was expressed at a much lower level in LR. Finally, we hypothesise that the poorly functionally characterised *SCL32* gene may play a part in the increased resistance to environmental stress of LR. We suggest that *BAM1*, *BFRUCT*, *EIL3*, *ERF5*, *ADT* paralogues (for functional redundancy), *PED1*, *GATA22‐like*, *HAT5‐like* and *SCL32* warrant further functional studies.

## Graphical abstract

We identified 22 candidate genes driving the phenotypic differences between fruits of *Lycium barbarum* and *Lycium ruthenicum*, known as goji berries, and studied their expression patterns during five developmental stages. The results helped us identify genes of particular interest for future studies of: (a) phenotypic traits important for the goji berry industry and (b) genes with unique or novel functions.

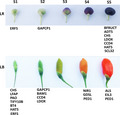

**Abbreviations**
CCAN: constitutive centromere associated complex
FSI: fruit shape index
LB: *Lycium barbarum*
LR: *Lycium ruthenicum*
SD: standard deviation

With the growing popularity of bioactive food, goji berries recently attracted almost global popularity both among scientists and consumers due to their high content of health‐promoting compounds, such as carotenoids, flavonoids, isoflavones and polysaccharide complexes [1, 2]. Goji berries have been used in China as food and medicine for millennia [1, 3], and a majority of the global commercial production still takes place in arid and semiarid areas of north‐western China [1]. Due to their high morphological similarity, fruits of two distinct species belonging to the family Solanaceae, *Lycium barbarum* (LB) and *Lycium chinense* (Chinese boxthorn), are usually marketed together as goji berries, but nearly 90% of all commercially available goji berries belong to the former species. A third closely related species, a wild perennial thorny shrub native to north‐western China, *Lycium ruthenicum* (LR; known as Russian box thorn, and its fruit as black wolfberry), has become a popular choice plant for combating soil desertification and for alleviating soil salinity/alkalinity due to its high resistance to the harsh environment of saline deserts [4, 5]. The fruit of this species, morphologically distinct from goji berries, is also popular in the traditional folk medicine, and there is some evidence that it may have even higher content of some bioactive compounds, such as flavonoids [6, 7, 8, 9]. More specifically, whereas the ripe LR fruit is deep purple in colour due to a high content of anthocyanins, the pigmentation of ripe LB fruit is reddish due to the high accumulation of carotenoids [9, 10, 11]. Regardless of this difference, with a growing amount of scientific evidence that consumption of both goji berries and black wolfberries has health benefits [1, 5, 6, 12], their popularity and economic importance is expected to continue growing.

Among the key questions in biology is the molecular basis for interspecies morphological diversity [13], but fruit phenotypes, and mechanisms by which they can be manipulated, have been in the focus of human interest long before the appearance of modern science, at least since we began domesticating fruit‐bearing plant species [14], and probably even much earlier [15, 16]. Evolutionary developmental biology is a research field focused on trying to understand the molecular mechanisms that control the development of an organism and orchestrate the emergence of phenotypic differences that allow organisms to survive in drastically different ecological niches around the planet [13], and last few decades of research have shown that transcriptional regulation during the development is a predominant mechanism for the generation of novel phenotypes [17, 18]. Despite the very close phylogenetic relationship of these two *Lycium* species [19, 20], their fruits are morphologically very different: as opposed to the red, elongated mature LB fruit, LR fruit is dark purple or black, round and smaller. Their metabolic profiles also differ significantly, particularly in the content of fatty acids, phenols and antioxidant capacities, which are much higher in LR, while the content of carotenoids, sugars, amino acids and osmolytes is higher in LB [6, 8, 9]. Although several studies have reported transcriptomic profiles of LB and/or LR fruits [7, 21, 22], fruit ripening is a complex developmental process, coordinated by a network of interacting genes and signalling pathways, and the genetic mechanisms underpinning these phenotypic differences remain only partially understood [6, 23, 24, 25, 26]. Therefore, the objective of our study was to study expression patterns of genes that may have major contributions to these phenotypic differences and identify individual genes that may have unique functions in any of these two species, and therefore warrant further studies. As transcriptional regulators play a central role in evolution [18], we focused our attention on these types of genes. We sampled fruits of both species during five developmental stages, from young fruit (9–12 days old) to ripe fruit (35–45 days old), and extracted the total RNA from these samples. To identify the candidate genes with most strikingly different expression profiles between the two fruits during different ripening stages, we relied on transcriptomic data deposited in public databases and then studied their expression in our samples using qPCR. These results shall lay the foundation for future studies of the underlying genetic basis for phenotypic differences between these two species.

## Materials and Methods

### Sample collection, RNA extraction and cDNA library construction

Fruits of both species were collected between 1 July and 20 August 2018, from two locations, both of which belong to the same arid climate zone. *Lycium ruthenicum* fruits were collected from nine wild shrubs growing in the vicinity of Bayan Taolaisu Wooden, Ejina, Alxa, Inner Mongolia, China (38°38′49″N; 106°91′10″E): elevation is 1162 m, the average annual rainfall is < 40 mm, the average temperature in July and August is 26.3 °C, soil type is salinised meadow, and surface salinity is 1.11%. *Lycium barbarum* fruits were collected from nine 5‐year‐old cultured shrubs from the germplasm nursery of the Ningxia Academy of Agriculture and Forestry Science, Lu Hua Tai plantations, Yinchuan, Ningxia, China (41°84′86″N; 100°97′69″E): elevation is 1148 m, the average annual rainfall is < 150 mm, the average temperature in July and August is 23.4 °C, soil type is light sierozem, and surface salinity is 0.09%. Since the two species have slightly different and variable fruit ripening times, we roughly divided the ripening period into five stages in order to be able to perform pairwise comparisons of different ripening stages. We collected samples of both species in the following time‐windows after the flowering (anthesis): S1 – young fruit (9–12 days); S2 – green fruit (14–19 days); S3 – colouring fruit (20–26 days); S4 – immature fruit (30–37 days); and S5 – mature fruit (34–45 days). When we use the term ‘stage’ throughout the paper, we refer to these five sampled stages. Each sampling was conducted in the morning between 9 am and 10 am, from the south‐facing side of the tree, approximately from the same part of the same branch, with environmental temperature ranging between 20 and 23 °C. At each sampling time‐point, several fruits were collected from three trees of each species and 15 fruits mixed into a single sample. This was repeated three times, thus obtaining three biological replicates (1 sample = 3 trees, 3 replicates = 9 trees). All samples were frozen immediately in liquid nitrogen and stored at −80 °C.

All fruit samples were ground to a powder in liquid nitrogen, and total RNA was extracted following the standard TRIzol extraction protocol [27]. The extracted total RNA was treated with RQ1 DNase (Promega, Madison, WI, USA), and its quality and quantity then determined by measuring the absorbance at A260/A280 and A260/A230 using Smartspec plus (Bio‐Rad, Hercules, CA,USA) spectrophotometer. RNA integrity was further verified by agarose gel electrophoresis. cDNA library was synthesised using RevertAid™ First Strand cDNA Synthesis Kit (Thermo Scientific, Waltham, MA, USA) following the manufacturer’s protocol.

### Gene selection process and sequence analysis

**Fig. 1:**
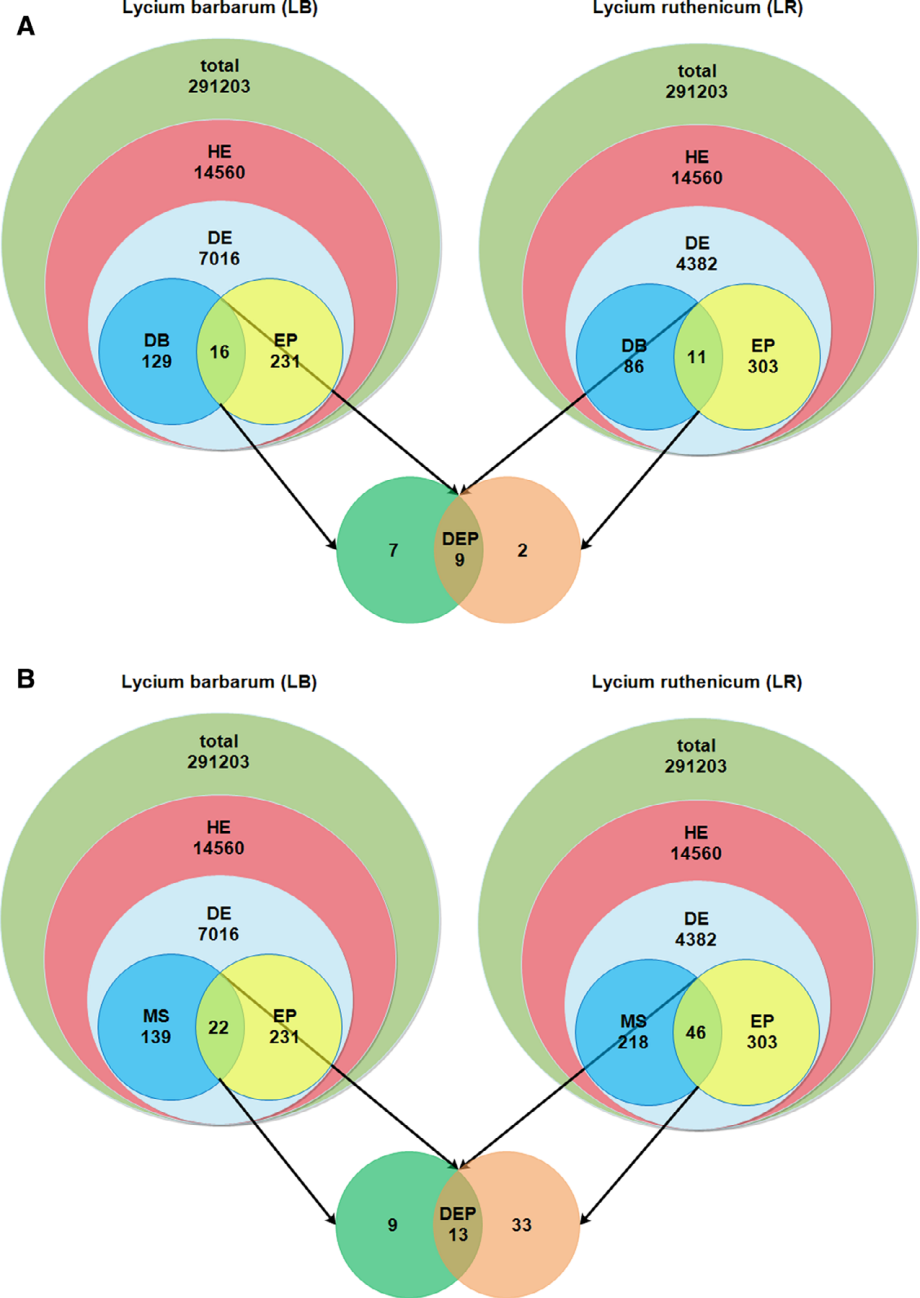
The two‐step selection process for genes analysed herein: (A) the selection of DNA‐binding genes, (B) the selection of genes by overlapping transcriptomic and metabolomics data. HE = highly expressed genes, DE = differentially expressed genes, DB = DNA‐binding genes, EP = genes peaking in each of the five developmental stages, MS = metabolome selection, DEP = genes overlapping between categories within species with pronouncedly different EP profiles between species (see text for detailed explanations).

Since transcriptional regulation is a predominant mechanism for the generation of novel phenotypes [17, 18], in order to study the molecular basis for morphological diversity between the fruits of these two species, we relied on available transcriptomic data for fruits of these two species to select a subset of (predominantly regulatory) genes that may have particularly large impacts on their phenotypic differences, using several series of selection criteria (Fig. 1). After retrieving the transcriptomic data for both species from the NCBIs GEO database (accession numbers GPL25820 and GPL25821), we merged the data to create a dataset of 291 203 genes. Among these, those with the mean FPKM across bioreplicates above the 95th percentile were selected as ‘highly expressed’ (HE) genes (14 560). From the HE dataset, genes with the differential expression q‐value between different development stages < 0.05 were selected independently for each species as a subset of ‘differentially expressed’ (DE) genes. Among the DE sets of genes, we selected target genes for qPCR via two rounds of selection. In the first round, we identified subsets of DNA‐binding (DB) genes and EP (‘expression peak’) genes and selected genes overlapping between categories within species with pronouncedly different EP profiles between species (DEP), thus identifying nine genes. In the second round, we overlapped EP genes with MS (‘metabolome selection’) genes in the same way to identify additional 13 genes for qPCR (Fig. 1; Appendix S1: Selection of genes tab). DB genes were identified using the InterProScan functional annotation tool in the custom GO database. EP genes were selected manually in a way to select genes with the expression peaking in each of the five developmental stages. MS category of genes was identified for each species in a previous study by overlapping the sets of differentially expressed genes and metabolites [22]. To annotate the identified ADT paralogues as reliably as possible, we conducted phylogenetic analyses using phylosuite [28] and its plug‐in programmes. For this analysis, we retrieved amino acid sequences of 59 homologues identified via BLASTx analysis. Sequences were aligned in mafft [29], poorly aligned segments removed using trimai [30], evolutionary model selected using modelfinder [31], and phylogenetic analysis conducted using the maximum likelihood algorithm implemented in IQ‐TREE [32]. Phylogram was visualised in itol [33].

### qPCR

Sequences from transcriptomic data were used as templates to design primers (Table 1) with the help of Primer3 web tool [34]. cDNA was diluted twofold and used as a template for qPCR, which was conducted on CFX Connect™ (BIO‐RAD), using Power SYBR® Green PCR Master Mix (Applied Biosystems® Cat: 4367659, Carlsbad, CA, USA). The PCR mix (50 µL) contained: 1.0 μL Ex taq, 25.0 μL 2X Ex taq Buffer (both Takara, Beijing, China), 1.0 μL (2.5 μm) dNTP Mix, 2 μL (10 μm) of each primer, 4.0 µL cDNA and 17.0 µL PCR‐Grade Water. Cycling conditions were as follows: 95 °C for 3 min, followed by 40 cycles of 95 °C for 10 s, primer pair‐specific TM (Table 1) for 20 s, 72.0 °C for 20 s and plate reading at 75 °C for 5 s. Melting curve was assessed from 65 to 95 °C, with 0.5 °C increments. We tested three reference genes (*18S*, *GAPDH* and *EF1α*) and selected *EF1α* as the most stable between the two species using NormFinder [35] and geNorm [36] (Appendix S1: Reference genes tab), which is in agreement with previous findings [4, 37]. Expression levels were calculated using the $2^{-\Delta \Delta C_{T}}$ method [38]. Biological and technical replicates were triplicate. Significance of differences in expression levels was tested using Fisher’s LSD test in originpro 8 (Star GmbH, Berlin, Germany; significance threshold set at 0.05).

**Table 1:** Primers used for qPCR. TM is the melting temperature (°C), amp is the size of the amplicon, and * marks the reference gene.

Gene	5′‐3′ sequences (F and R)	TM	amp
*BT4*	AGCTCGGACTAAGAATGTTGACT	59.49	154
	TGAGGAGGAGGAGACAGAACA	59.57	
*GDSL*	TGGTCCGACATGCTTCCTTC	60.04	127
	TCCCTGTGAGAAACCTTGGC	59.89	
*SCL32*	CCTGCAAGCTCTGTCACACT	60.25	129
	TCAGCGACTCACATCATGGT	59.10	
*GATA22*	CCTGCTCCTCGTGTTGAATCA	60.34	149
	GCAGCTTCCTTCTCATCCTGT	60.07	
*DXS2*	TACGCTACATCGAACGCTCC	59.97	122
	GGCTATGATCAGGTGGTGCA	59.82	
*HAT5*	GCCGTCTTACTCCTGAGCAG	60.18	136
	TTTTGGAACCACACAGCCAC	59.18	
*TIFY10B*	CGGCCCTTTCGCAAACTCTA	60.67	169
	TTCTGATCGAGCGTCCATCA	58.90	
*GAPCP1*	AAGGAGCGAGACAGTTGGTG	59.97	178
	TTTGTGGACAGAGAGGGTGC	59.89	
*ADT5‐X1*	ATTGCACACGAACTCTGCCA	60.53	142
	TCAACCCGAATCGCCTCATT	59.75	
*ADT5‐X2*	ACCGTATAGATCAGCTGCGC	60.04	138
	CTAACTCTCACAAAGCTCGGC	59.00	
*BAM1*	CGAGGACAAGGGCGTTAAGA	59.75	139
	CATCTGGGCAATGGGAAGGT	60.03	
*BFRUCT*	AATCTTGCTTACCCCGCCAA	59.96	146
	CCATTTTGAGGCCCAGTCCA	60.25	
*PED1*	GCCACAACAACCTTCTGTCT	58.32	172
	ACCAAAATACAGGGGTCTTCC	57.55	
*ALS*	GGGGCCTCCTTTTAGTTTCA	57.40	149
	TCCACAAATTCCACCACCAGA	59.50	
*NIR1*	GCCCTGAATCCTTCTACACCC	60.13	173
	GCGAAACGATGTGATGAGGC	59.97	
*Lignin‐forming anionic peroxidase*	CTCCAGCCAAGACAACTCCAA	60.20	156
	TTGCAAGTGAGAAAGCTGCAC	59.93	
*LDOX*	ACCTCCGGCAACCTTAACAC	60.25	172
	AGGACCTCAAGTACCGACGA	59.96	
*Primary amine oxidase*	CAGAGCTTGCGATTCCTGGA	60.11	142
	ACAGTGACTTTGATGCTTCCAC	59.12	
*CHS*	CATTCGAGCCCTTCACCAGT	60.04	153
	GTTGGCCCTAAAACCGGAGA	59.96	
*CCD4*	CGTCTAGGCAAGGTGGAAGG	60.11	173
	TCCCTCCGTTATTTTCAATGCA	58.31	
*EIL3*	CAGGAGGAAGTCTTGGCTCG	60.11	131
	ATAGGGTCATCAACGGCACC	59.82	
*ERF5*	CCGTCGATTTCAAGCTCCGA	60.46	122
	TCGAACTTCTTCACCCGAGG	59.40	
EF1α*	CCATACCAGCATCACCATTCTTC	59	117
	GTCACACTTCCCACATTGCC	59	

## Results and Discussion

We compared the fruit morphology and expression of 22 genes in developing fruits of *L. barbarum* and *ruthenicum*.

### Fruit morphology

**Fig. 2:**
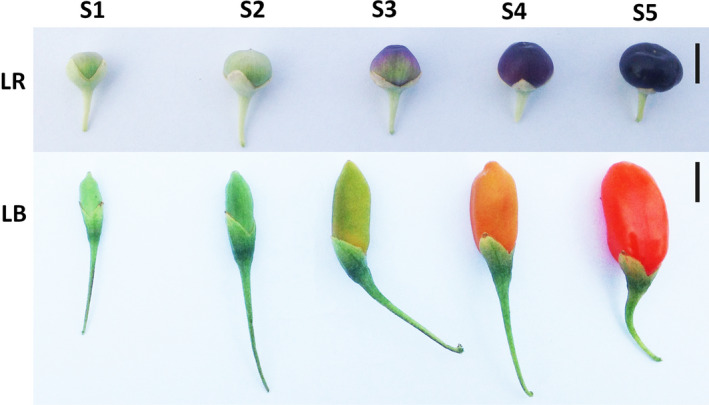
Images of *Lycium barbarum* (LB) and *Lycium ruthenicum* (LR) fruits at five developmental stages (S1–S5). Scale bars represent 5 mm.

Fruit ripening was divided into five developmental stages (S1 to S5) according to the fruit phenotype (Fig. 2, Table 2). *Lycium ruthenicum* fruits in both S1 and S2 were green, aside from the appearance of light pink hues in the latter stage, but their shapes differed; S3 was the colour‐breaking stage, marked by the spreading of pink and light purple hues; S4 fruits were purple; and S5 fruits were dark purple, almost black (Fig. 2). *Lycium barbarum* fruits in both S1 and S2 were green, but their shapes differed; S3 was the colour‐breaking stage, marked by the spreading of yellow colour; S4 fruits were orange; and S5 fruits were red (Fig. 2). Whereas FSI values did not exhibit a pronounced temporal trend in either species, differences in the remaining morphometric parameters exhibited increasing temporal profiles. Fruits of the two species differed in shape from the first sampled stage (9–12 days postflowering), and these differences mostly increased during the maturation. LB fruits exhibited a reduction in length between S3 and S4. This can be explained by a strong increase in the cheek diameter during this period, also reflected in the FSI inflection point.

**Table 2:** Morphology of *L. barbarum* and *ruthenicum* fruits during five developmental stages. Developmental stages were defined as: S1 – young fruit (9–12 days postflowering); S2 – green fruit (14–19 days); S3 – colouring fruit (20–26 days); S4 – immature fruit (30–37 days); and S5 – mature fruit (34–45 days). Length and cheek were both measured as diameters. FSI is fruit shape index = length diameter/cheek diameter. Values are presented as averages of 25 fruits ± standard deviation (SD).

Stage	*Lycium barbarum*				*Lycium ruthenicum*			
	Mass (g)	Length (mm)	Cheek (mm)	FSI	Mass (g)	Length (mm)	Cheek (mm)	FSI
S1	0.05 ± 0.01	7.94 ± 1.06	3.43 ± 0.29	2.33 ± 0.36	0.03 ± 0.00	3.65 ± 0.45	4.41 ± 0.32	0.83 ± 0.10
S2	0.14 ± 0.03	12.68 ± 1.26	4.31 ± 0.39	2.96 ± 0.37	0.05 ± 0.01	4.25 ± 0.58	5.07 ± 0.75	0.84 ± 0.07
S3	0.21 ± 0.07	14.66 ± 2.61	4.94 ± 0.53	2.99 ± 0.55	0.08 ± 0.02	4.42 ± 0.55	5.32 ± 0.37	0.83 ± 0.09
S4	0.26 ± 0.07	14.34 ± 2.41	5.78 ± 0.93	2.55 ± 0.62	0.11 ± 0.04	4.82 ± 0.66	5.74 ± 0.84	0.84 ± 0.08
S5	0.48 ± 0.18	15.64 ± 3.03	7.39 ± 0.93	2.12 ± 0.39	0.13 ± 0.04	5.05 ± 0.58	5.96 ± 0.77	0.85 ± 0.06

### Gene expression

We divided the 22 selected genes into six functional categories: glycolytic energy production and sugar metabolism, fruit ripening and ethylene, anthocyanin biosynthesis, carotenoid metabolism, stress responses (jasmonate signalling), growth and development, and regulation of transcription, development and growth (Table 3). Due to functional complexity of many of these genes, some of them could be simultaneously assigned to more than one category, so this categorisation should be treated as merely provisional.

**Table 3:** The 22 studied genes, provisionally divided into six functional categories. ‘‐l’ stands for ‘‐like’, LFAP is lignin‐forming anionic peroxidase, and PAO is primary amine oxidase.

Category	Glycolytic energy production and sugar metabolism	Carotenoid metabolism	Fruit ripening and ethylene	Stress responses (jasmonate signalling), growth and development	Anthocyanin biosynthesis	Regulation of transcription, development and growth
Genes	GAPCP1 BAM1 BFRUCT	DXS2 CCD4	ALS PED1 NIR1 TIFY10B LFAP PAO	EIL3 ERF5	ADT5‐X1 ADT5‐X2 CHS LDOX	GATA22‐l BT4 GDSL‐l HAT5‐l SCL32

### Glycolytic energy production and sugar metabolism

#### Glyceraldehyde‐3‐phosphate dehydrogenase A, chloroplastic (GAPCP1)

**Fig. 3:**
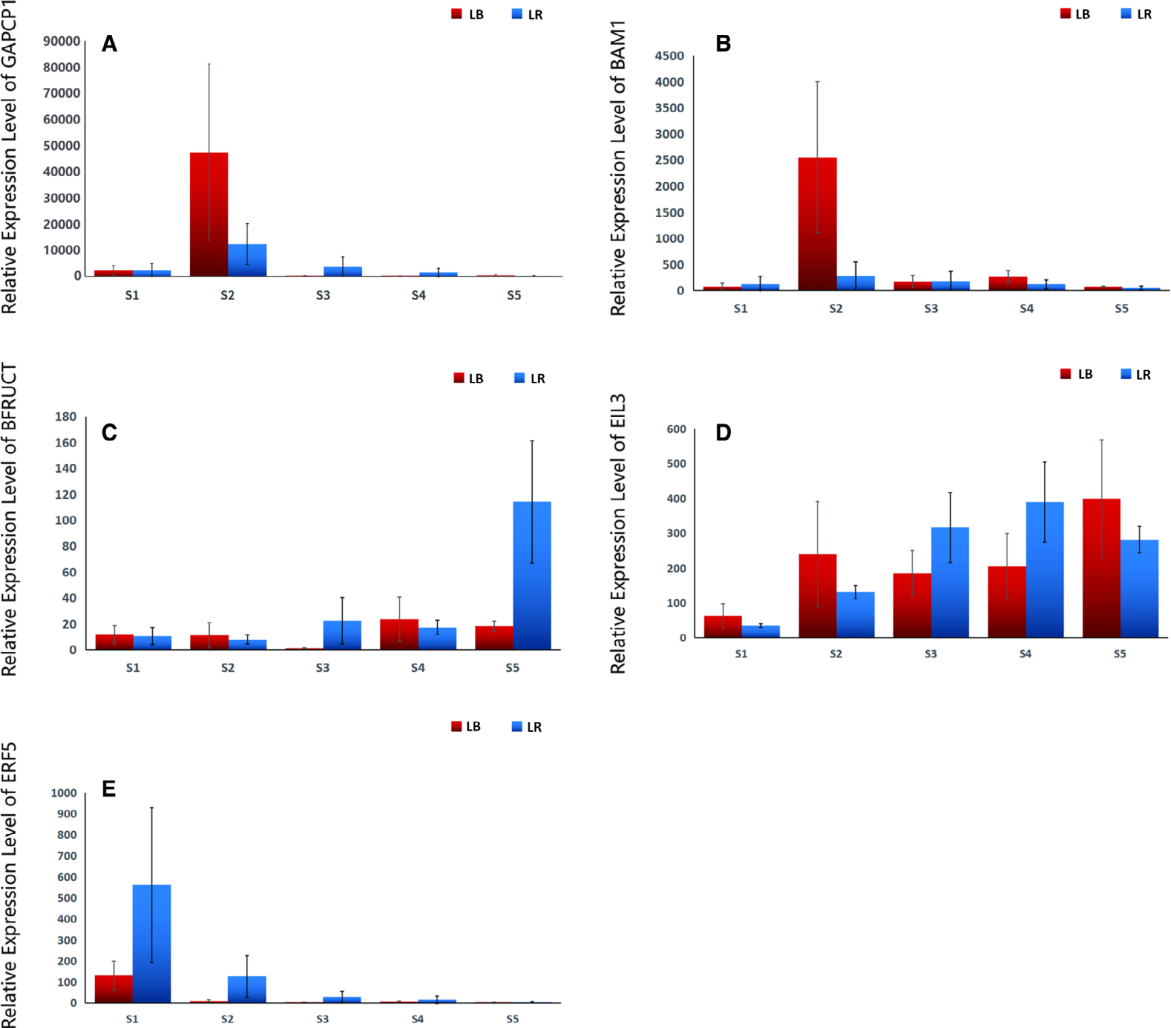
Expression patterns of genes putatively associated with glycolytic energy production and sugar metabolism (A–C), and fruit ripening and ethylene: (A) *GAPCP1*, (B) *BAM1*, (C) *BFRUCT*, (D) *EIL3*, (E) *ERF5*. LB represents *Lycium barbarum*, LR represents *Lycium ruthenicum*, S1 to S5 (*x*‐axis) represent five sampled developmental stages, and data are presented as average relative expression level ± SD (*y*‐axis).

*GAPCP1* expression exhibited very high upregulation in the 2nd stage in both species, but much higher in LB (albeit with a large SD) (Fig. 3A, Appendix S1). This gene plays a major role in glycolytic energy production in nongreen plastids and chloroplasts, and it is essential for breakdown of starch to form sucrose for export to nonphotosynthetic tissues and to generate primary metabolites for anabolic pathways such as fatty acid and amino acid synthesis [39, 40, 41]. As such, it has an important developmental role, for example in pollen development [39], and in the production of glycolytic energy in fruits [41]. We hypothesise that its high expression in the S2 may be associated with its role in maintaining the sugar and amino acid balance, especially the serine biosynthesis [39, 40].

#### Beta‐amylase 1 (BAM1)

This gene also exhibited a strong upregulation in the 2nd stage, but only in LB (also with a large SD) (Fig. 3B). BAM1 is a major enzyme in starch breakdown and maltose metabolism in chloroplasts during the night, but it has complex and only partially understood functions [42]. The expression pattern observed in our study may be associated with the higher sugar content in LB, so specific roles of this gene may be of interest for the selection and breeding programmes of this cultured species.

#### Beta‐fructofuranosidase (BFRUCT)

This gene exhibited a strong upregulation in ripe LR fruit (5th stage) (Fig. 3C). Genes from this relatively large family are generally associated with sugar metabolism (galactose, starch and sucrose), but they have broad substrate specificity [43]. Importantly, it has been identified as a putative candidate gene for regulating the vitamin C, sugar and acid content in fruit under water deficit conditions [44]. Therefore, high expression of this gene in ripe LR fruit is likely to be an evolutionary adaptation to fruit ripening in arid environment. This finding warrants further functional studies of BFRUCT in LR.

### Fruit ripening and ethylene

#### Ethylene‐insensitive protein 3 (EIL3)

In both species, it exhibited high expression throughout the studied period, with slowly increasing temporal expression profile, peaking in 4th stage in LR and in ripe fruit in LB (albeit with relatively high SD values in some stages) (Fig. 3D). It is a probable transcription factor acting as a positive regulator in the ethylene response pathway, crucial for fruit ripening [45]. It binds a primary ethylene response element present in the *ethylene response factor1* promoter, thus activates the transcription of this gene and initiates the ethylene‐mediated downstream transcriptional cascade [45, 46]. Therefore, its high expression in the final ripening stages in *Lycium* fruits (Fig. 3D) is not surprising. However, in other plants it also has positive roles in the anthocyanin content regulation [45] and abiotic (salt and drought) stress resistance [47], so it should not be excluded that its continuously high expression in these two species a reflection of their adaptation to arid environments.

#### Ethylene‐responsive transcription factor 5 (ERF5 or EREBP)

It followed a relatively similar pattern in both species: very high upregulation in the 1st stage, followed by a strong downregulation in the 2nd stage and relatively low expression afterwards (Fig. 3E). The upregulation was consistently much higher in LR. Similar to the previous gene, *ERF5* is also a transcriptional activator that promotes ripening and colouring in fruit [23], but its expression pattern in the two *Lycium* species is not in agreement with this function. As it has also been implicated in drought (abiotic stress) responses [48], it is much more likely that it plays a role in abiotic stress resistance in the young fruit. Further functional studies are warranted.

### Anthocyanin biosynthesis

#### Arogenate dehydratases (putatively ADT5)

There are six *ADT* paralogues in the *Arabidopsis* genome and designated as *ADT1*–*6* (some studies also use the name prephenate dehydratase, PD or PDT). They are believed to be functionally redundant and associated with plant growth and biosynthesis of anthocyanin, the natural pigment of plants, responsible for red, blue and purple colours [49]. Two *Lycium ADT* paralogues passed our screening process, but we identified seven putative ADT fragments in the transcriptomic data. As the identification of some of them was difficult, we conducted a phylogenetic analysis (Supplementary file: ADT panel). This analysis revealed that many of the previously studied ADT genes were misannotated. The structure of the phylogram suggests the existence of six to seven clades, but some of them remain paraphyletic, and they do not correspond to the six paralogues described in *Arabidopsis*. Putting this issue in order is beyond the scope of our study, but our analyses indicate that *ADT1* and *ADT2* form two well‐supported, monophyletic and distinct sister clades, whose duplication appears to have occurred rather deep in the evolutionary history. *ADT4* forms another monophyletic clade, which contains numerous misannotated genes (*ADT5* and *6*). Nested between these two is a paraphyletic clade which we provisionally named *ADT3*. Further studies are needed to assess the orthology of these genes, many of which are also misannotated. The topology of the remaining clades is poorly resolved. Following previous studies, we named the clade that contained the two isoforms whose expression we studied here, *ADT5*. However, in this way, ADT6 clade is nested between the clades ADT4 and ADT5, which is not the most logical solution. The status of a handful of highly derived sequences at the end of the phylogram also remains questionable; they could represent a part of a large catch‐all ADT5 clade or a new ATD7 clade. Alternatively, the catch‐all clade could be named ADT6, as nominal ADT6 sequences are scattered throughout the phylogram. We identified seven fragments corresponding to *ADT* genes in the expressed gene libraries, but alignment indicates that these fragments identified as *ADT1* probably belong to only two paralogues, which would add up to six expressed ADT paralogues in these two *Lycium* species. Following the above outlined logic, three fragments were unambiguously identified as *ADT1* (X1‐3) and one as *ADT2*. One paralogue was named *ATD3*, although the clade is paraphyletic, and topology indicates that it may be ancestral to *ADT1* and *2*. As mentioned above, on the basis of previously annotated *ADT5* genes in a number of *Capsicum* species, we named the two orthologues studied here *ADT5‐X1* and *X2*, but this clade may as well be renamed into ADT6 by future studies.

**Fig. 4:**
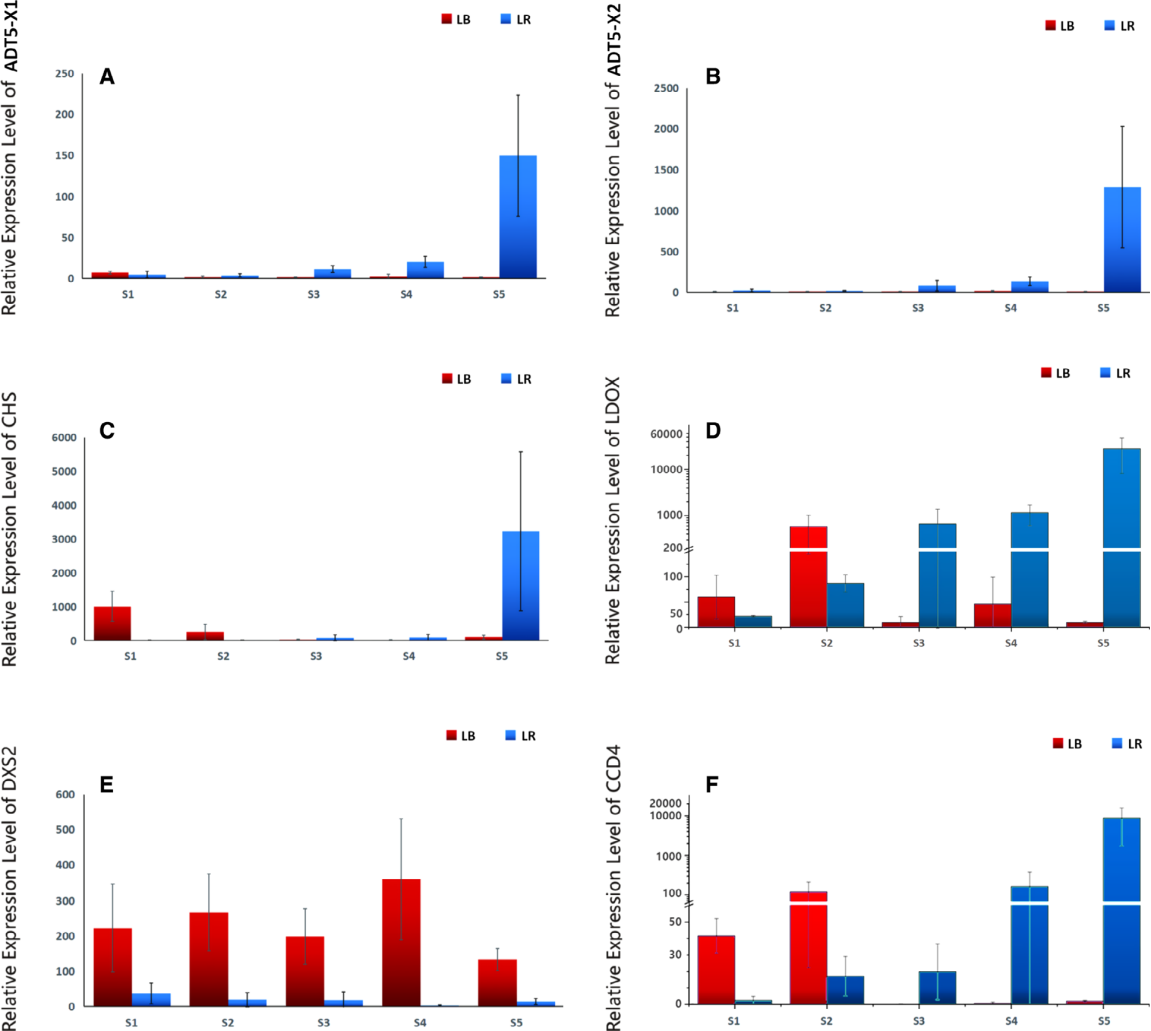
Expression patterns of genes putatively associated with anthocyanin biosynthesis (A–D) and carotenoid metabolism (E–F): (A) *ADT5‐X1*, (B) *ADT5‐X2*, (C) *CHS*, (D) *LDOX*, (E) *DXS2*, (F) *CCD4*. LB represents *Lycium barbarum*, LR represents *Lycium ruthenicum*, S1 to S5 (*x*‐axis) represent five sampled developmental stages, and data are presented as average relative expression level ± SD (*y*‐axis).

As regards the expression patterns of these two paralogues, they were rather similar, with a very strong upregulation in the ripe LR fruit (Fig. 4A,B). In LB, the expression of both paralogues was low throughout the studied period, with a peak in the 1st stage. In LR, the expression of both paralogues was low in the first two stages, followed by a continuously sharply increasing profile during the last three stages. The expression of the *ADT5‐X2* was continuously higher, especially so in the ripe fruit, where it was ten orders of magnitude higher than that of the *ADT5‐X2*. Therefore, the expression patterns observed in our study are in perfect agreement with high anthocyanin content in LR [6, 7, 8, 9], but they indicate that the *ADT5‐X2* isoform may play a more pronounced role in the accumulation of anthocyanins in LR. It would be interesting to assess via functional studies whether these two isoforms are fully functionally redundant in LR.

#### Chalcone synthase 1 (CHS)

Expression patterns of this gene exhibited different trajectories in the two studied species (Fig. 4C). In LB, it exhibited a U‐shaped trajectory, very high upregulation in the 1st stage, low expression in the 3rd stage and again rather strong upregulation in the ripe fruit. In LR, its expression was very low in the first two stages, upregulated in 3rd and 4th stages and then exceptionally highly upregulated in the ripe fruit. This gene is crucial for anthocyanin synthesis, and this is not the first report of its high upregulation during fruit maturation in LR [7].

#### Leucoanthocyanidin dioxygenase (LDOX)

This gene is also known as anthocyanidin synthase (ANS1). In LR, it exhibited a continuously high and increasing expression, with exceptionally strong upregulation in ripe fruit (Fig. 4D). Its expression in LB was variable, with a strong upregulation in the 2nd stage and mild upregulation in the 4th stage. This gene also takes part in the biosynthesis of anthocyanin [50, 51, 52], and it is commonly upregulated in later stages of fruit ripening [25, 51].

In the light of much higher anthocyanin content in LR fruits [6, 8, 9], we can conclude that all four of these genes (two ADT paralogues*, CHS* and *LDOX*) are likely to be crucial elements of the mechanism via which the fruits of LR accumulate higher levels of anthocyanin than LB fruits. However, high expression of *CHS* and *LDOX* in early stages in LB indicates that these two genes may possess other functions in this species, which warrants further functional studies in LB.

### Carotenoid metabolism

#### 1‐deoxy‐d‐xylulose 5‐phosphate‐synthase 2 (DXS2)

During all five stages, *DXS2* was highly expressed in LB (Fig. 4E). In LR, its expression was highest in the 1st stage and lowest in the 4^th^ stage. This gene is known to be associated with carotenoid biosynthesis; for example, in melon, two *DXS2* paralogues (a and b) are induced in flowers and ripening fruit of orange‐fleshed, but not white‐fleshed, varieties, which perfectly coincides with β‐carotene accumulation [8]. This is also in agreement with high accumulation of carotenoids in LB [9, 10, 11], reflected in its reddish colour (Table 2). Our results exhibit partial congruence with the report of Liu *et al*. [9], who found that in LR *DXS2* was expressed more highly in the early than in late fruit ripening stages, whereas in LB its expression exhibited a bell‐shaped curve (low in early and late stages, but high in the middle stages, around the colour‐breaking stage). *DXS1*, a divergent homologue of *DXS2*, also associated with carotenoid biosynthesis in plants [9, 53], did not pass our screening process for highly regulated genes, which indirectly supports the finding that this gene is much more highly expressed in the leaves than in the fruit of *Lycium* species [9]. Therefore, multiple lines of evidence suggest that *DXS2*, but not *DXS1*, plays a crucial role in the accumulation of carotenoids in *Lycium* fruits.

#### Carotenoid cleavage dioxygenase 4 (CCD4)

This gene exhibited opposite temporal expression patterns in the two species (Fig. 4F). In LB, high expression in the first two stages was followed by an almost complete absence of expression in the last three stages. In LR, this gene exhibited a continuous increase in expression, from very low in the first stage to exceptionally high in ripe fruit. *CCD4* plays a decisive role in the regulation of the carotenoid content in plants (and fruit) by degrading the carotenoids into colourless compounds [54]. Much higher expression levels in LR (compared to LB) have been observed before [9]. Therefore, our results indicate that much higher content of carotenoids in LB [6, 8] is a consequence of both higher accumulation in LB during all developmental stages (via *DXS2*; Fig. 4E) and strong degradation of carotenoids in LR in the final stage of fruit ripening (via *CCD4*; Fig. 4F).

### Stress responses (jasmonate signalling), growth and development

Several genes could be associated both with stress responses and regulation of growth and development, so their classification is somewhat ambiguous.

#### Lignin‐forming anionic peroxidase

**Fig. 5:**
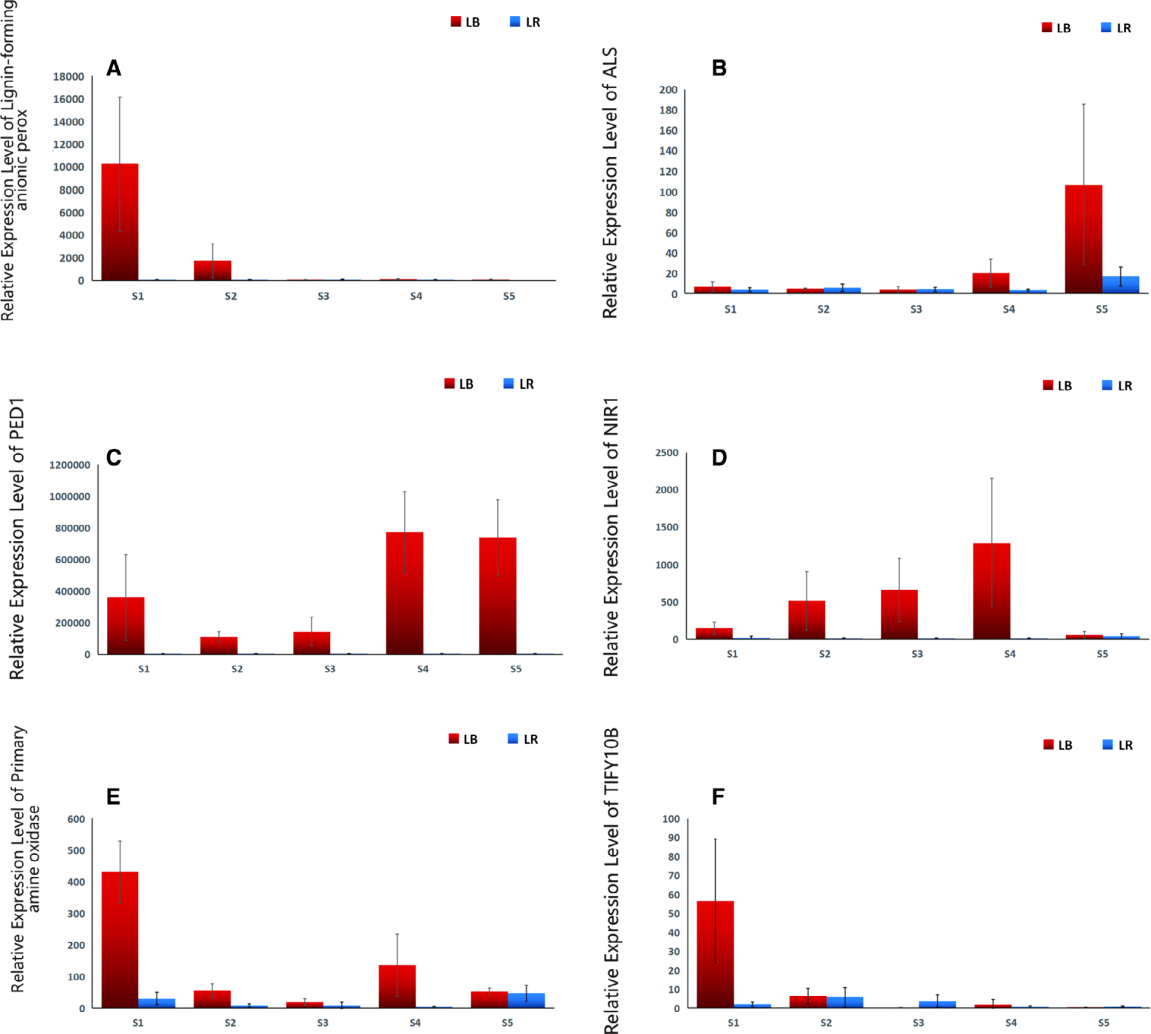
Expression patterns of six genes putatively associated with stress responses (jasmonate signalling), growth and development: (A) *lignin‐forming anionic peroxidase*, (B) *ALS*, (C) *PED1*, (D) *NIR1*, (E) *primary amine oxidase*, (F) *TIFY10B*. LB represents *Lycium barbarum*, LR represents *Lycium ruthenicum*, S1 to S5 (*x*‐axis) represent five sampled developmental stages, and data are presented as average relative expression level ± SD (*y*‐axis).

This gene was extremely highly upregulated in LB in the 1st stage, followed by a strong decrease in the 2nd stage (but still very high expression), and then low expression in the remaining stages (the uptick in the 4th stage has high SD, so this may be attributed to molecular noise) (Fig. 5A). In LR, expression was low throughout, with weak upregulation in ripe fruit. This gene has a broad scope of roles in the organism, including the removal of H_2_O_2_, oxidation of toxic reductants, auxin catabolism and regulation of stress responses to both biotic and abiotic stressors [55, 56, 57]. Furthermore, it plays an integral role in the secondary cell wall biosynthesis, and biosynthesis and degradation of lignin, by forming cross‐links between cellulose, pectin, hydroxyproline‐rich glycoproteins and lignin [58]. Our results indicate that it may play a crucial role in the cell wall biosynthesis in the early developmental stages in LB and that its comparatively high expression may be reflected in the higher growth rate of fruits of this species.

#### Acetolactate synthase (ALS, also SuRB)

It exhibited similar expression profiles in both species, low in early stages, with an increase in ripe fruit. In LB, weak upregulation was observed in the 4th stage, followed by a relatively strong upregulation in the 5th stage (with high SD value) (Fig. 5B). In LR, there was only a mild upregulation in the 5th stage. This gene catalyses the formation of acetolactate from pyruvate, the first step in valine and isoleucine biosynthesis, so much higher expression levels in LB might be associated with the higher growth rate of this species (Table 2). However, as valine and particularly isoleucine are important for cold stress resistance [59], strong regulation of this gene may also be related to cold stress [60], possibly due to a wide gap between daily temperature maximums and minimums in deserts.

#### 3‐ketoacyl‐CoA thiolase 2, peroxisomal (PED1)

The expression of this gene was exceptionally high in LB throughout the studied period, whereas in LR its expression was comparatively low throughout the first four stages, with a mild upregulation in the ripe fruit (Fig. 5C). In LB, the expression peaked in the last two stages. This gene is involved in the long chain fatty acid beta‐oxidation during germination and subsequent seedling growth in *Arabidopsis* [61], but it is also involved in the jasmonic acid biosynthesis during senescence (strong upregulation during leaf senescence) [62]. Jasmonates are signalling molecules involved in stress responses (such as salt stress) [63], but also development and secondary metabolism biosynthesis. Therefore, its expression pattern in LR, observed in this study, is in agreement with its role in senescence, but it is not clear why the expression is so much higher in LB. This indicates that the gene might have different functions in the two species and warrants further functional studies in LB.

#### Ferredoxin–nitrite reductase (NIR1)

In LB, this gene exhibited a high expression throughout the studied period, with a temporally increasing profile, peaking in the 4th stage, followed by a sharp drop in ripe fruit (Fig. 5D). In LR, the expression was mostly low, with a minor upregulation in the 1st and 5th stages. *NIR1* is involved in transport and reduction of nitrites, which are tied to growth and development, as nitrogen is an important constituent of a huge number of critical plant compounds [64]. However, this gene is also involved in the jasmonic acid signalling pathway and upregulated by stress [64]. For example, natural defences against herbivory are initiated by leaf damage (e.g. chewing by insects or vertebrates) and mediated by activation of the jasmonic acid (JA) signalling pathway. We hypothesise that its relative abundance in LB is associated with its higher growth rate.

#### Primary amine oxidase

In LB, the expression of this gene was exceptionally high in the first stage, followed by intermediate expression in the remaining stages (upregulation in 4th stage, but with very high SD) (Fig. 5E). In LR, the expression levels were lower, exhibiting minor peaks in 1st and 5th stages (with high SDs). This gene oxidises the aliphatic diamine putrescine into aldehyde, ammonia and hydrogen peroxide, and it has varied and complex functions in the organism, both in key developmental processes and stress responses. It may also be involved in the regulation of developmental programmed cell death. Importantly, it is also required for jasmonic acid‐mediated early development in roots [65].

#### Protein TIFY 10B (TIFY10B)

The expression of this gene was mildly upregulated in LB in the first stage (but with a large SD), followed by very low expression in the following four stages. In LR, it exhibited very low expression throughout the studied period (Fig. 5F). This gene, also known as *jasmonate ZIM domain‐containing protein 2*, is a repressor of jasmonate responses [66, 67]. The conserved TIFY motif within the ZIM domain mediates interactions between most jasmonate ZIM domain proteins [66]. In strawberry, most *TIFY* genes exhibited a decrease in expression from early fruit development to ripening stages; as this coincides with a decrease in the accumulation proanthocyanidins (flavonols), this suggests that jasmonate metabolism might have a key role in the synthesis of proanthocyanidins [68]. Although other previous studies mostly associated *TIFY10B* with abiotic stress responses (drought and salt) [63, 69], due to antioxidant and antistress properties of proanthocyanidins [70], these two roles are not mutually exclusive.

### Regulation of transcription, development and growth

#### GATA transcription factor 22‐like (GATA22‐like)

**Fig. 6:**
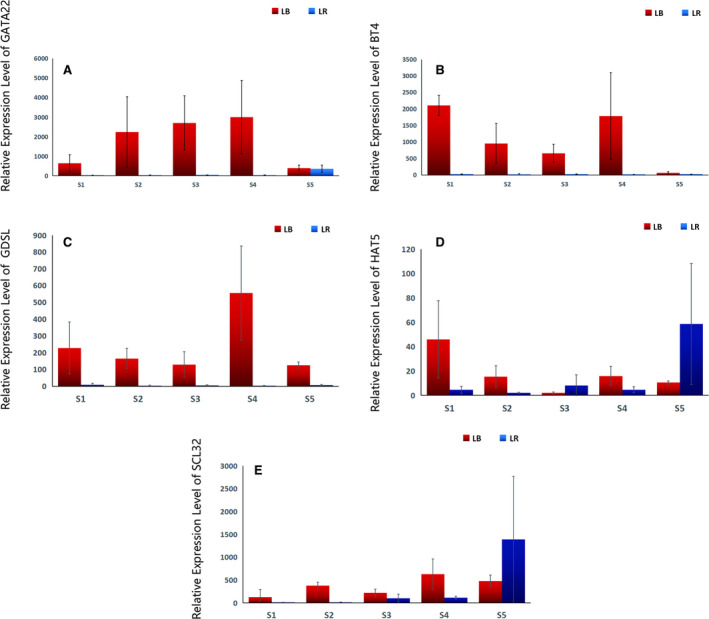
Expression patterns of six genes putatively associated with regulation of transcription, development and growth: (A) *GATA22*, (B) *BT4*, (C) *GDSL*, (D) *HAT5*, (E) *SCL32*. LB represents *Lycium barbarum*, LR represents *Lycium ruthenicum*, S1 to S5 (*x*‐axis) represent five sampled developmental stages, and data are presented as average relative expression level ± SD (*y*‐axis).

In LB, the expression of this gene exhibited an increasing temporal profile during the first four stages, followed by a sharp drop‐off in ripe fruit, but its expression levels were very high throughout the studied period (Fig. 6A). In LR, it was constitutively expressed, with a strong upregulation in ripe fruit. *GATA22* is a transcriptional regulator that specifically binds 5'‐GATA‐3' or 5'‐GAT‐3' motifs within gene promoters. It is a regulator of germination, senescence, elongation growth and flowering time. It is involved in the modulation of chloroplast development, promotes chlorophyll biosynthesis and modulates growth and division in a cytokinin‐dependent manner, and it is a repressor of the gibberellic acid signalling pathway, regulates flowering and modulates greening during the flowering, and it is also involved in the regulation of sugar‐sensing genes and influences leaf starch content [71, 72]. Our results indicate that in LB fruit, it may play a more important role in growth than in ripening, whereas in LR it appears to have much more important role in ripening than in growth. More studies are needed to confirm this.

#### BTB/POZ and TAZ domain‐containing protein 4 (BT4)

The expression of *BT4* was continuously very high during the first four stages in LB (with some variation and rather high SD values in S2 and S4), but it exhibited a strong downregulation in the in ripe fruit (Fig. 6B). In LR, it was expressed at comparatively low levels during all five stages. Functionally, this is also a transcription regulator (zinc finger) that belongs to a family of genes with major roles in plant development via its role in the ubiquitination and subsequent proteasomal degradation of target proteins [73].

#### GDSL esterase/lipase At4g01130‐like (GDSL‐like)

This gene was also expressed at a very high level in LB during all five stages, with a peak in the 4th stage (but with a high SD) (Fig. 6C). Its expression was very low in LR throughout the studied period. GDSL esterases/lipases are a subclass of lipolytic enzymes with multifunctional properties, involved in the regulation of plant development, morphogenesis, synthesis of secondary metabolites and defence responses [74]. Higher expression in LB of these two transcription regulation factors associated with development and growth (*BT4* and *GDSL‐like*) is likely to be associated with faster growth and larger size of LB fruit (Table 2).

#### Homeobox‐leucine zipper protein HAT5‐like (HAT5‐like, also known as ATHB1)

This gene exhibited opposite expression patterns in the two species (Fig. 6D). In LB, the expression of this gene was relatively high only in the 1st stage. In the remaining three stages, the expression was relatively low, with very low expression in the 3rd stage. In LR, expression was low during the first four stages (lowest in the 2nd stage), with a relatively strong upregulation in the 5th stage. It belongs to the large HD‐zip family of transcription factors with diverse roles in developmental processes and adaption to abiotic stress in plants [75, 76]. This is not the first indication of its key role in fruit development regulation, likely to be mediated by hormone accumulation in fruit tissue [77]. However, opposite expression patterns in LB and LR suggest that this gene may have different functions in these two species. As LB exhibits higher growth rate than LR, higher expression in LB the first stage may be associated with its higher growth rate; as LR undergoes higher levels of abiotic stress (somewhat higher soil salinity and lower rainfall in our experimental setup), its upregulation in ripe LR may be associated with abiotic stress resistance. As this is purely speculative, further studies are needed to identify causes for this upregulation in ripe LR.

#### SCL32 (scarecrow‐like protein 32‐like)

This gene also exhibited different expression profiles between the two species (Fig. 6E). In LB, we observed a continuously high expression (with relatively high SD values). In LR, it exhibited a temporally increasing profile, with very low expression in the 1st stage, low in the 2nd, highly upregulated in 3rd and 4th stages and very high upregulation in the 5th stage (albeit with a very high SD). Its expression was higher in LB during the first four stages, but in ripe fruit it was much higher in LR. This is very intriguing as *SCL32* remains functionally largely uncharacterised [78]. It belongs to the large plant‐specific GAI‐RGA‐and‐SCR (GRAS) gene family, members of which are transcriptional regulators that play critical roles in development and signalling. For example, they are involved in gibberellic acid (gibberellin) and phytochrome signalling, which regulate various aspects of plant growth and development [78, 79, 80]. It has been hypothesised that *SCL32* is a probable transcription factor involved in plant development and regulation of transcription [78], and there is indirect evidence that it may be involved in the floral transition [81] and mycorrhisation [82]. *SCL32* has been identified as a RIN target (MADS box transcription factor ripening inhibitor). RIN is one of the earliest acting ripening regulators, required for both ethylene‐dependent and ethylene‐independent ripening regulatory pathways, which appears to regulate carotenoid biosynthesis as well [83]. Therefore, higher expression in LB than in LR during the first four studied developmental stages may be explained by its roles in growth regulation and carotenoid biosynthesis, both of which are more pronounced in LB (Table 2). However, this does not explain its sudden upregulation in ripe LR fruit, whereas its expression in ripe LB fruit was comparable to that in other four stages. This indicates a unique function for this gene in the ripe LR.

Intriguingly, another gene from the same family, *SCL7*, has been associated with root growth and tolerance to abiotic stress: seedling stage *Arabidopsis* overexpressing *SCL7* exhibited faster root growth and significantly increased tolerance to abiotic stress (slower rate of water loss) [84]. SCL7 protein localises to the nucleus of the plant cell, which is consistent with its putative role as a transcriptional regulator [84]. This gene was also identified in *Populus euphratica*, the most important species for large‐scale afforestation projects on saline desert sites in China, that can tolerate exceptionally high salt concentrations [84]. As *L. ruthenicum* is native to the salinised deserts of Northwest China, it generally exhibits a higher resistance to abiotic and biotic stressors common in that environment, such as high soil salinity, drought and local pests, than *L. barbarum*. Therefore, its genetics and physiology should bear strong markings of the evolution in an environment where drought and salt stress are very common. We could not identify *SCL7* among the differentially expressed genes, so we hypothesise that *SCL32* may have taken over the function of resistance to environmental stress of in LR. An indirect support for this hypothesis may come from *Euphorbia esula*, a perennial weed that is considered glyphosate tolerant, where *SCL32* exhibited increased expression in response to glyphosate [85]. Future studies should further explore the expression and functions of this gene in *L. ruthenicum* and its putative role in the abiotic stress resistance in plants.

## Conclusions

The interpretation of our findings is somewhat complicated by different environmental parameters at the two sampled locations, as well as by the fact that LB has undergone generations of anthropogenic selection for higher growth, whereas the genome of LR is likely to be shaped solely by nonanthropogenic factors. As a result, it is difficult to disentangle the genetic from environmental variables and anthropogenic from nonanthropogenic variables. Another limitation of our study is high interindividual variability in gene expression levels (high SD values) observed for many samples. While a relatively high level of molecular noise is common in gene expression studies [86, 87], high interindividual variability in gene expression was particularly pronounced in genes that are involved in environmental responses in *Arabidopsis thaliana* [88]. This is an indication that harsh environmental conditions may be causing pronouncedly volatile gene expression levels on the interindividual, or possibly even intraindividual, levels on relatively short time scales, but this hypothesis warrants further studies. In particular, to assess the extent of this variability, future studies should attempt to use a larger number of biological replicates. However, the objective of this study was not to infer gene functions with confidence, but merely identify genes that may be of interest to us and other teams aiming to conduct functional genetic studies on these two species in the future. We identified a number of genes (transcription factors) that may be the crucial drivers behind the notable phenotypic differences between LB and LR fruits and singled out several genes that warrant further studies. Notably, *PED1* might have different functions in the two species. *BAM1* may be contributing to higher sugar content in LB, so specific roles of this gene may be of interest for the selection and breeding programmes of this cultured species. And finally, we hypothesise that as yet functionally noncharacterised gene, *SCL32*, may play a part in the increased resistance to environmental stress of *L. ruthenicum*.

## Conflict of interest

The authors declare no conflict of interest.

## Author contributions

JZ, HL and YC conceived and designed the work. YL, YF, HL, YY and WA acquired the samples and conducted wet laboratory experiments. JZ, XQ and YW analysed and interpreted the data. JZ drafted the manuscript. All authors revised it critically for important intellectual content and approved the final version.

## Supporting information

Appendix S1. qPCR raw data, reference genes, and selection procedure.

## Data Availability

All relevant data are provided within the article and supplementary files.
